# Central Corneal Thickness and Endothelial Cell Changes in Diabetics and Age-Matched Non-diabetics in a Tertiary Care Hospital in Central India

**DOI:** 10.7759/cureus.57234

**Published:** 2024-03-30

**Authors:** Sachin Daigavane, Vijaya Mallareddy

**Affiliations:** 1 Ophthalmology, Jawaharlal Nehru Medical College, Datta Meghe Institute of Higher Education and Research, Wardha, IND

**Keywords:** comparative study, ophthalmology, endothelial cells, central corneal thickness, corneal morphology, diabetes

## Abstract

Background

Diabetes has become an epidemic, significantly impacting ocular health as one of its end-organ responses. Among the various ocular complications, alterations in corneal morphology stand out. Central corneal thickness (CCT) and endothelial cell function are vital parameters in assessing intraocular pressure, conducting pre-refractive surgery evaluations, and maintaining corneal transparency. Understanding these changes in diabetic individuals compared to non-diabetics is crucial for managing ocular health in this population.

Aim and objective

This study evaluates and compares CCT and endothelial cell changes between diabetic individuals and age-matched non-diabetics. By analyzing these parameters, the study seeks to provide insights into the impact of diabetes on corneal morphology and its implications for ocular health.

Methods

The study recruited 124 patients from the Ophthalmology Outpatient Department (OPD) at Acharya Vinoba Bhave Rural Hospital (AVBRH), Sawangi. A cross-sectional research design was employed to collect data over six months. Patients were carefully selected, and informed consent was obtained from all participants. CCT and endothelial cell parameters were assessed using specular microscopy, a non-invasive imaging technique. Statistical analysis was done using the software Statistical Package for the Social Sciences (SPSS) for inferential statistics, such as t-tests and ANOVA, and comparing parameters between diabetic and non-diabetic groups. Findings were interpreted based on both statistical significance and clinical relevance.

Results

In diabetic patients, the mean CCT was 547.91 µm, while it was 523.62 µm in non-diabetic individuals. The T statistic for this variable was 5.14, indicating a ^17^ significant difference between the two groups. Similarly, significant differences were found between diabetics and non-diabetics for endothelial cell density, coefficient of variation, and hexagonality, as evidenced by their respective T statistics of 7.46, 5.17, and 4.91. Endothelial cell density averaged 2375 cells/mm^2^ in diabetics and 2666.95 cells/mm^2^ in non-diabetics. Additionally, the coefficient of variation was higher among people with diabetes (40.87%) compared to non-diabetics (35.09%). Hexagonality, a measure of endothelial cell shape, was lower in diabetic corneas (40.48%) than in non-diabetic corneas (46.46%).

Conclusion

The study observed significant differences in corneal morphology, including central thickness and endothelial cell changes, between diabetic and non-diabetic individuals. These findings underscore the impact of diabetes on ocular health and emphasize the importance of monitoring corneal parameters in diabetic patients. Understanding these changes can aid in better management and treatment strategies for ocular complications associated with diabetes.

## Introduction

Diabetes mellitus poses a significant global health concern with far-reaching systemic implications. Projections indicate that by 2030, the number of individuals affected by diabetes will soar to 643 million [1]. It is estimated that diabetes impacts approximately 6.4% of the global population [2]. The ocular manifestations of diabetes encompass a spectrum of conditions, including diabetic retinopathy, diabetic macular edema, extraocular muscle palsy, dry eye, diplopia, neovascular glaucoma, refractive changes, progression of cataract, and ischemic optic neuropathy [2]. Numerous studies have concluded that diabetic corneas often exhibit increased central corneal thickness (CCT) and decreased endothelial cell density (ECD), alongside evidence of polymegathism and polymorphism [3]. These changes impact ocular health and have implications for various procedures such as cataract surgery, refractive surgery, and keratoplasty, where the cornea's condition plays a crucial role [4].

Metabolic stress induced by hyperglycemia alters corneal endothelial cells, leading to reduced hexagonality and increased variability [5]. Advanced glycosylation end products further exacerbate corneal thickening by fostering covalent bonds, effectively acting as cross-linking agents [6]. Additionally, advanced glycation end products and matrix metalloproteinases can instigate corneal damage through endothelial cell apoptosis [7]. High glucose levels also interfere with Na-K+ ATPase activity, a critical component of endothelial cells, resulting in morphological changes that affect the permeability of diabetic corneas [8].

Moreover, endothelial cell count naturally declines with age, leading to an adult range of 2000-3000 cells/mm^2^, significantly lower than the count at birth, which typically falls within the 4000-5000 cells/mm^2^ [9,10]. Understanding corneal morphological parameters becomes pivotal as they provide valuable insights into the overall health status of the cornea [11]. Specular microscopy is instrumental in capturing endothelial cells, facilitating subsequent analysis through computer programs [12]. This technique allows for high-magnification visualization, aiding in detecting endothelial damage [13]. Additionally, specular microscopy is a valuable tool for measuring CCT, boasting advantages such as ease of operation [14].

## Materials and methods

Study setting, design, and duration

The study was conducted at the Department of Ophthalmology within Acharya Vinoba Bhave Rural Hospital (AVBRH), Sawangi, utilizing a cross-sectional research design. Its objective was to investigate CCT and endothelial cell changes in individuals with diabetes compared to age-matched non-diabetic counterparts. The standardized protocol was implemented for horizontal specular microscopy to measure a mean. The measurements were performed manually by ophthalmologists. Data was collected and analyzed over a meticulously executed six-month period from November 2022 to May 2023. One hundred and twenty-four patients participated in the study, divided into two groups: 62 known cases of diabetes and 62 non-diabetic individuals. These participants were carefully selected from individuals seeking medical attention at the hospital during the study period.

Participants

The study included patients attending the Ophthalmology Outpatient Department (OPD) at AVBRH. Informed consent was obtained from all participants before their inclusion in the study. Each subject's right eye was primarily examined unless ocular pathology necessitated the assessment of the left eye.

Inclusion criteria

Patients aged 40 years and above were eligible for inclusion. All individuals presenting to the Ophthalmology OPD were considered for participation and were categorized as diabetics or non-diabetics based on their medical history.

Exclusion criteria

Patients below the age of 40 were excluded from the study. Additionally, individuals with existing corneal diseases such as opacities or dystrophy, pterygium, entropion, or trichiasis were ineligible. Diagnosed cases of glaucoma or pterygium, cataract, pseudophakics, and refractive surgery and those with dry eye disease or a history of contact lens wear were also excluded. Moreover, participants who were unwilling or unable to provide informed consent were excluded from the study.

Data collection procedure

The data collection procedure commenced with recruiting patients attending the Ophthalmology OPD at AVBRH, Sawangi, for potential participation in the study. Criteria such as age above 40 years and the absence of specific eye conditions were applied to identify suitable candidates. Following patient identification, eligible individuals were provided with a thorough explanation of the study's objectives, procedures, and potential risks and their rights as participants. Informed consent was obtained from those who voluntarily agreed to participate and signed the consent form. Subsequently, a baseline assessment was conducted for each participant to gather demographic information, medical history (including diabetes status), and other relevant clinical data. This preliminary evaluation aided in categorizing participants into diabetic and non-diabetic groups based on their medical backgrounds. Trained ophthalmologists then performed comprehensive ophthalmic examinations to assess various parameters related to corneal morphology.

These examinations included the measurement of CCT, ECD, coefficient of variation (CV), and hexagonality. Horizontal specular microscopy was performed by ophthalmologists; it was a non-invasive imaging technique, employed to capture high-resolution images of the corneal endothelium and measure CCT accurately. This technique facilitated the precise quantification of endothelial cell parameters and provided insights into the participants' corneal health status. During the examination process, data recording was carried out meticulously for each participant, capturing relevant information such as CCT measurements, ECD counts, and morphological characteristics of corneal endothelial cells. Standardized protocols were followed to ensure consistency and accuracy in data collection across all participants.

After the data collection phase was completed, statistical analysis was performed using the appropriate software Statistical Package for the Social Sciences (SPSS). Descriptive statistics, such as means, standard deviations, and ranges, were calculated to summarize the collected data. Inferential statistics, such as t-tests or ANOVA, were employed to compare parameters between diabetic and non-diabetic groups, providing valuable insights into potential differences in corneal morphology associated with diabetes. Throughout the data collection process, strict adherence to ethical guidelines was maintained to protect participants' rights, uphold confidentiality, and ensure compliance with ethical standards governing human subjects this comprehensive approach to data collection aimed to gather accurate and reliable data for subsequent analysis and interpretation.

Ethical considerations

The study prioritized ethical principles by obtaining informed consent from participants, ensuring confidentiality, and upholding the right to withdraw. Approval from the Datta Meghe Institute of Medical Sciences Institutional Ethics Committee was obtained to maintain ethical standards (approval number: DMIMS(DU)/IEC/2022/414).

Statistical analysis

Data analysis involved descriptive statistics summarizing participant characteristics and inferential statistics, such as t-tests or ANOVA, comparing parameters between diabetic and non-diabetic groups. Statistical significance was determined using p-values, typically set at 0.05, and conducted using appropriate software like SPSS to ensure accuracy. Findings were interpreted based on both statistical significance and clinical relevance.

## Results

Table 1 describes the distribution of patients by age and gender, focusing on diabetes status. It showed the number and percentage of diabetic and non-diabetic individuals across age groups (40-49, 50-59, 60-69, and 70+ years) and genders (male and female).

**Table 1:** Patient distribution into two groups according to their demographic variables

Variable	Diabetics		Non-diabetics	
	Frequency	Percentage (%)	Frequency	Percentage (%)
Age				
40-49 years	1	1.61%	0	0%
50-59 years	11	17.74%	9	14.52%
60-69 years	23	37.10%	22	35.48%
≥70 years	27	43.55%	31	50%
Total	62	100%	62	100%
Gender				
Male	38	61.29%	34	54.84%
Female	24	38.71%	28	45.16%
Total	62	100%	62	100%

Table 2 presents data from a past study comparing diabetics (n=62) and non-diabetics (n=62). It included variables such as CCT, ECD, CV, and hexagonality. For CCT, the mean thickness for diabetics was 547.91 µm, with an SD of 23.87 and a standard error of 3.03, while for non-diabetics, the mean was 523.62 µm, with an SD of 28.47 and a standard error of 3.61. The T statistic for this variable was 5.14, indicating a significant difference between the two groups. Similarly, significant differences were found between diabetics and non-diabetics for ECD, CV, and hexagonality, as evidenced by their respective T statistics of 7.46, 5.17, and 4.91. These findings suggest distinct corneal characteristics between individuals with and without diabetes in the study population.

**Table 2:** Comparison of corneal characteristics between diabetics and non-diabetics CCT: corneal central thickness; ECD: endothelial cell density; CV: coefficient of variation

Variable	Diabetics (n=62)			Non-diabetics (n=62)			T statistic
	Mean	SD	Standard error	Mean	SD	Standard error	
CCT (µm)	547.91	23.87	3.03	523.62	28.47	3.61	5.14
ECD (cells/mm^2^)	2375	230.85	29.31	2666.95	203.59	25.85	7.46
CV (%)	40.87	6.72	0.85	35.39	5.64	0.71	5.17
Hexagonality (%)	40.48	5.29	0.67	46.46	7.98	1.01	4.91

## Discussion

Analyzing corneal parameters such as CCT, ECD, CV, and hexagonality across both study groups revealed significant statistical disparities between individuals with diabetes and those without. Specifically, diabetic subjects exhibited heightened CCT, diminished corneal ECD, elevated CV, and reduced hexagonality. According to Wiemer et al. [15], CCT experiences an increase irrespective of the severity of retinopathy in diabetic patients. This suggests that changes in corneal thickness may occur independent of other diabetic complications, highlighting the complexity of diabetic ocular pathology.

Conversely, Storr-Paulsen et al. [16] noted a notable rise in CCT without any differences in ECD, CV, and hexagonality between diabetic and non-diabetic cohorts. This discrepancy in findings underscores the variability in corneal parameters observed among diabetic populations, possibly influenced by factors such as disease duration, glycemic control, and individual patient characteristics. Similarly, Roszkowska et al. [17] reported statistically significant reductions in ECD coupled with augmented CCT among diabetic individuals compared to their non-diabetic counterparts, further highlighting the multifaceted nature of diabetic corneal changes.

Furthermore, Lee et al. [18] observed heightened CCT, diminished ECD, and elevated CV in diabetic patients compared to non-diabetics. Their findings suggest a progressive deterioration of corneal endothelial function associated with diabetes, which may have implications for long-term ocular health outcomes in diabetic individuals. Notably, individuals with diabetes enduring over a decade exhibited endothelial cell modifications characterized by increased CCT, higher CV, lower ECD, and reduced hexagonality. This underscores the importance of considering disease duration and its potential impact on corneal parameters in diabetic patients.

In contrast, Inoue et al. [19] found a decreased CV and ECD in diabetic patients. At the same time, no significant differences in hexagonality and CCT were observed compared to non-diabetic counterparts. These findings suggest that certain corneal parameters may remain relatively unaffected in diabetic individuals, highlighting the heterogeneity of diabetic ocular manifestations. Sudhir et al. [20] reported lower corneal ECD in diabetic subjects than in non-diabetics, with no notable changes in CCT, hexagonality, or CV observed between the two study groups. This emphasizes the importance of comprehensive assessment and monitoring of corneal health in diabetic patients to identify potential changes that may necessitate intervention.

Limitations

The study's limitations include small sample size, short duration, lack of consideration for disease duration and HbA1c levels, single-center setting, and potential biases. These factors may restrict the generalizability of findings and limit the study's ability to capture long-term trends in corneal parameters among diabetic and non-diabetic individuals. Addressing these limitations in future research could lead to a more comprehensive understanding of corneal changes associated with diabetes.

## Conclusions

Our study highlights significant differences in corneal morphology among diabetic individuals compared to non-diabetics. Specifically, diabetic patients exhibit alterations in CCT, ECD, CV, and hexagonality. These findings underscore the importance of monitoring corneal health in diabetic patients and emphasize the need for further research to understand better the implications of these morphological changes on ocular health outcomes in this population.
